# Clinical relevance of AI-based PD-L1 scoring in non-small cell lung cancer

**DOI:** 10.3389/fonc.2026.1790571

**Published:** 2026-04-07

**Authors:** Mateusz Maniewski, Jędrzej Borowczak, Justyna Durślewicz, Marek Zdrenka, Adam Kowalewski, Łukasz Szylberg

**Affiliations:** 1 Department of Obstetrics, Gynecology and Oncology, Collegium Medicum in Bydgoszcz, Nicolaus Copernicus University in Torun, Bydgoszcz, Poland; 2 Department of Tumor Pathology and Pathomorphology, Oncology Centre Prof. Franciszek Łukaszczyk Memorial Hospital, Bydgoszcz, Poland; 3 Faculty of Medicine, Bydgoszcz University of Science and Technology, Bydgoszcz, Poland; 4 Clinical Department of Oncology, Oncology Centre Prof. Franciszek Łukaszczyk Memorial Hospital, Bydgoszcz, Poland

**Keywords:** AI-based algorithm, artificial intelligence (AI), computational pathology, digital pathology, non-small cell lung cancer (NSCLC), PD-L1 scoring algorithm, predictive biomarkers

## Abstract

Artificial intelligence (AI)-based algorithms are increasingly implemented in histopathological cancer diagnostics to enhance reproducibility and efficiency in biomarker assessment. Although AI-driven image analysis shows promise in standardizing immunohistochemical evaluation and reducing inter-observer variability, its clinical reliability as a substitute for expert assessment requires rigorous validation. Minor discrepancies in Programmed Death Ligand 1 (PD-L1) interpretation can alter patient stratification and treatment outcomes, especially in borderline expression ranges. This study evaluated the concordance between PD-L1 expression scores assessed by an expert pathologist and the uPath VENTANA PD-L1 (SP263) Assay Algorithm. The cohort included 333 non-small cell lung cancer (NSCLC) cases stained with the anti-PD-L1 (SP263) antibody. Digital slides were independently assessed by a board-certified pathologist and the uPath algorithm. Analysis used the Tumor Proportion Score (TPS), stratified into three categories: <1%, 1–49%, and ≥50%. The overall concordance of PD-L1 status (cutoff ≥1%) between the uPath algorithm and the pathologist was 95.2% (κ = 0.89), while the concordance for the three-tier categorization was 94.5% (weighted κ = 0.93). The median PD-L1 expression score from uPath was 1.2 percentage points (pp) higher than the pathologist’s assessment. Discrepancies varied by TPS group, with mean differences of 0.2 pp, 4.4 pp, and 1.95 pp for TPS <1%, 1–49%, and ≥50%, respectively. Category changes occurred in 6% (20/333) of cases, potentially altering immunotherapy eligibility. AI-assisted assessment demonstrates high concordance with the expert pathologist, particularly in binary classification. However, due to discrepancies in the intermediate range, the algorithm is currently best positioned as a supportive tool for verifying borderline cases.

## 1 Introduction

The immunohistochemical (IHC) expression of programmed death ligand 1 (PD-L1) in tumor cells serves as a key predictive marker for immunotherapy in non-small cell lung cancer (NSCLC) (1). Assessment of PD-L1 requires substantial expertise, ongoing validation, and continuous training by pathologists, owing to the variety of available antibody clones (e.g., SP263, 22C3, SP142, 28-8), differing IHC platforms (Agilent and Ventana Medical Systems Inc., Tucson, AZ, USA), and complex scoring systems (2–7).

In NSCLC, treatment decisions hinge on PD-L1 status, determined by the pathologist using the Tumor Proportion Score (TPS). This straightforward metric represents the percentage of PD-L1-positive tumor cells relative to all viable tumor cells in a sample. Clinically relevant cutoffs are 1% and 50%, categorizing cases as PD-L1-negative (TPS <1%), PD-L1-positive (TPS 1–49%), or high PD-L1 expression (TPS ≥50%) (1, 8, 9).

Advancements in digital pathology have enabled the integration of artificial intelligence (AI) tools into histopathological diagnostics. AI-assisted algorithms can mitigate intra- and inter-observer variability, accelerate time to treatment, and assist less experienced pathologists. Commercially available options include research-use-only (RUO) algorithms and certified products (FDA-approved or CE-IVD marked) (10, 11). Although studies highlight the advantages of AI in pathology, uncertainties persist regarding its clinical implementation (12, 13).

This study compares PD-L1 assessments by a board-certified pathologist trained in Roche scoring guidelines with an AI-assisted algorithm in NSCLC cases from a tertiary pathology department at the Certified European Lung Cancer Centre.

## 2 Material and methods

### 2.1 Materials and sample acquisition

This retrospective study analyzed 333 formalin-fixed, paraffin-embedded (FFPE) cases collected from routine diagnostic procedures at the Department of Tumor Pathology and Pathomorphology, Oncology Center in Bydgoszcz, Poland, between July 2021 and March 2025. The study was conducted as a retrospective technical validation of the AI-based algorithm. Clinical diagnostic and treatment decisions for the patients were made prior to this study based on the original pathology reports. The results of this study did not influence diagnostic or treatment decisions for patients. Clinical data, including age, sex, race, TNM status, tumor grade, histology, and type of collected specimen were collected. TNM staging was available mostly for post-operative cases, leading to a high proportion of Tx cases. Patients characteristic are summarized in **Table 1**. Sections (3 μm thick) were prepared from each FFPE block and mounted on glass slides for histopathological and IHC evaluation. The study included preoperative core-needle biopsy specimens and postoperative surgical resection specimens. Cytological specimens were excluded.

**Table 1:** Patient characteristics (n = 333).

Clinical data		n (%)
Samples		333
Median age		69 years (IQR 64-73)
Age categories	<65 years	88 (26.4%)
	65–74 years	186 (55.9%)
	75–84 years	56 (16.8%)
	>85 years	3 (0.1%)
Sex	Female	140 (42%)
	Male	193 (58%)
Race	Caucasian	333 (100%)
Type of material	Biopsy specimen	189 (56.8%)
	Resection specimen	144 (43.2%)
Grade	G1	15 (4.5%)
	G2	195 (58.6%)
	G3	89 (26.7%)
	Gx	34 (14.7%)
Stage	T1	70 (21%)
	T2	15 (4.5%)
	T3	13 (3.9%)
	T4	9 (2.7%)
	Tx	226 (67.8%)
Lymph nodes invasion	N0	86 (25.8%)
	N1	17 (5.1%)
	N2	11 (3.3%)
	N3	2 (0.6%)
	Nx	217 (65.2%)
Histopathology	SCC	127 (38.1%)
	Non-SCC	196 (58.9%)
	Mixed type	10 (3%)
Pleural invasion	0	92 (27.6%)
	1	31 (9.3%)
	2	5 (1.5%)
	3	2 (0.6%)
	Not assessed	203 (61%)

TNM staging was available only for post-operative cases; therefore, a high proportion of Tx cases is observed.

### 2.2 Immunohistochemical staining

**Figure 1:**
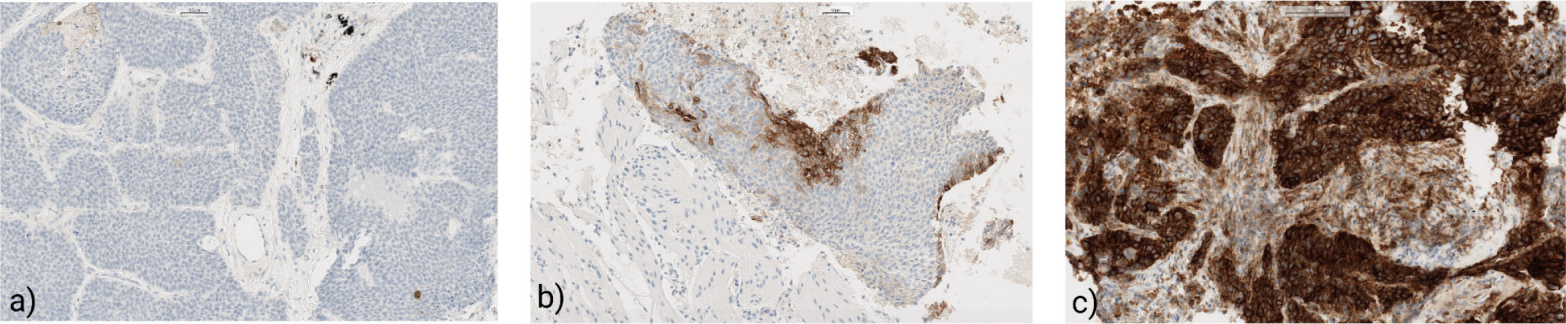
Immunohistochemical patterns of PD-L1 expression stained by the SP263. **(a)** Negative PD-L1 expression in NSCLC (0,1% TPS); **(b)** Positive PD-L1 expression in NSCLC (15% TPS); **(c)** High-PD-L1 expression in lung cancer (98% TPS).

PD-L1 expression was assessed via IHC using the VENTANA PD-L1 (SP263) assay (Roche/Ventana). Tissue sections underwent deparaffinization, rehydration, and antigen retrieval with Ventana’s cell conditioning solution per manufacturer guidelines. The primary antibody (rabbit monoclonal anti–PD-L1 clone SP263) was applied on the fully automated Ventana BenchMark ULTRA staining platform. Detection employed the OptiView DAB IHC Detection Kit, followed by hematoxylin counterstaining. Each run included positive and negative external controls to verify technical quality (**Figure 1**).

### 2.3 PD-L1 scoring (tumor proportion score)

**Figure 2:**
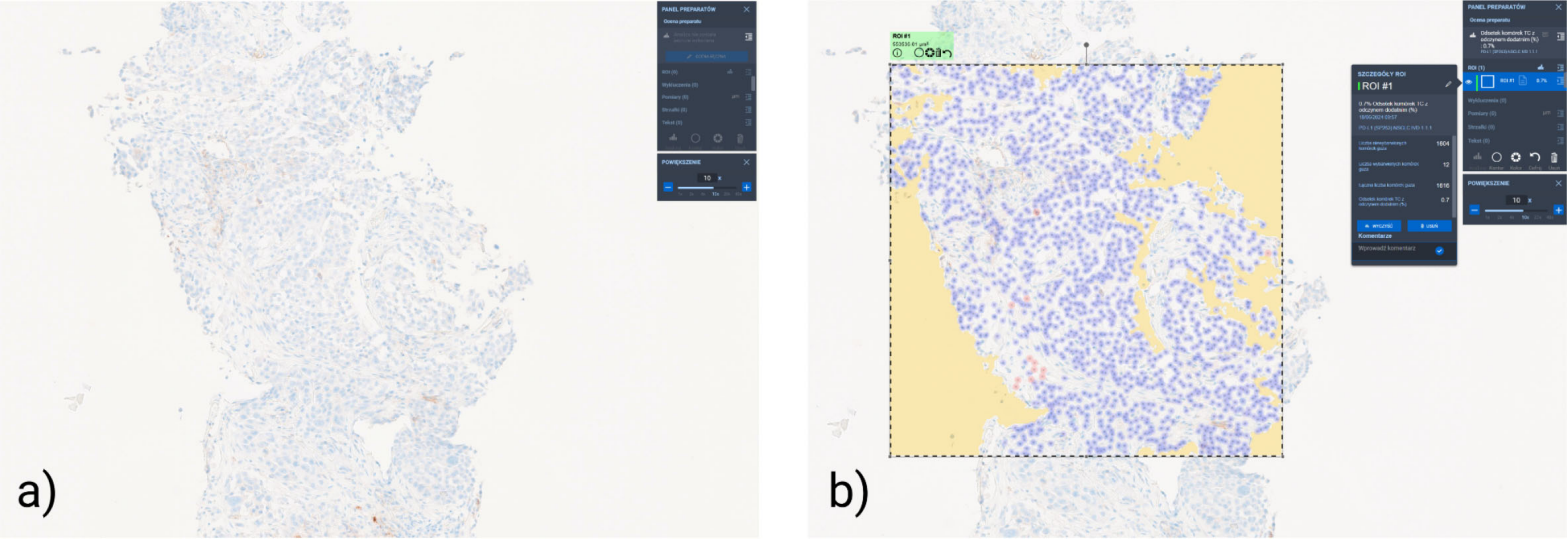
Digital image analysis of PD-L1 expression (clone SP263) in non-small cell lung cancer (NSCLC) using the uPath software. **(a)** Representative part of whole-slide image (WSI) of a PD-L1-stained NSCLC section demonstrating the raw immunohistochemical (IHC). **(b)** Corresponding visualization after manual Region of Interest (ROI) selection and subsequent uPath digital pathology algorithm TPS assessment.

TPS was defined as the percentage of viable tumor cells exhibiting partial or complete membranous staining for PD-L1, irrespective of intensity. Evaluations were performed independently via two methods. Manual scoring involved microscopic review by an experienced, board-certified pathologist (M.Z.) (blinded to clinical data and algorithm results), who estimated the proportion of PD-L1-positive tumor cells among all viable tumor cells and recorded TPS as a percentage (0–100%). Concurrently, slides were digitized using the Ventana DP 600 digital scanner (Roche Diagnostics) at 40× magnification to produce whole-slide images (WSI). AI-assisted scoring utilized the PD-L1 image analysis algorithm in Roche Ventana uPath Digital Pathology software (**Figure 2**). The regions of interest (ROIs) were selected during routine diagnostics by an experienced pathologist, with the number (1-3) and location of ROIs determined by specimen size and the presence of viable cell fields. Necrotic and non-tumor areas were excluded. The algorithm automatically detected tumor cells, classified them by membranous PD-L1 staining, and computed TPS. Quality control measures excluded artifacts and non-tumor regions per manufacturer protocols. Outputs included visual markups of PD-L1–positive cells and the corresponding TPS values.

Both assessments were conducted blindly and independently, with TPS values recorded for comparative analysis.

### 2.4 Statistical analysis

Due to the nature of the expression data, statistical analyses were performed using nonparametric tests. Differences in PD-L1 expression between subgroups were analyzed using the Mann-Whitney test or Kruskal-Wallis test. Concordance was measured with Cohen’s κ and weighted κ. Statistical significance was set at P < 0.05. Analyses were performed using Microsoft Excel 2021 and Statistica v 13.3 (StatSoft).

## 3 Results

Agreement between the expert pathologist and the uPath algorithm was high across all metrics. For binary PD-L1 status (TPS <1% vs. ≥1%), overall concordance reached 88.8% with κ = 0.89 (95% CI: 0.83-0.94). Using the expert pathologist’s assessment as the reference standard, uPath achieved a sensitivity of 95.3% (95% CI: 91.7–97.6%) and a specificity of 95.0% (95% CI: 88.7–98.4%), with a positive predictive value (PPV) of 97.8%, a negative predictive value (NPV) of 89.6%, and an overall accuracy of 95.2%. At the clinically relevant TPS ≥50% threshold, classification performance further improved, with a sensitivity of 97.5% and specificity of 99.2%. Both PPV and NPV exceeded 97%, resulting in an overall accuracy of 98.8% (**Table 2**).

**Table 2:** Performance of uPath relative to expert assessment at clinically relevant TPS thresholds.

Threshold	Sensitivity % (95% CI)	Specificity % (95% CI)	PPV % (95% CI)	NPV % (95% CI)	Accuracy %
≥1%	95.3 (91.7–97.6)	95.0 (88.7–98.4)	97.8 (95.0–99.1)	89.6 (82.9–93.9)	95.2
≥50%	97.5 (91.4–99.7)	99.2 (97.2–99.9)	97.5 (90.9–99.4)	99.2 (97.0–99.8)	98.8

**Figure 3:**
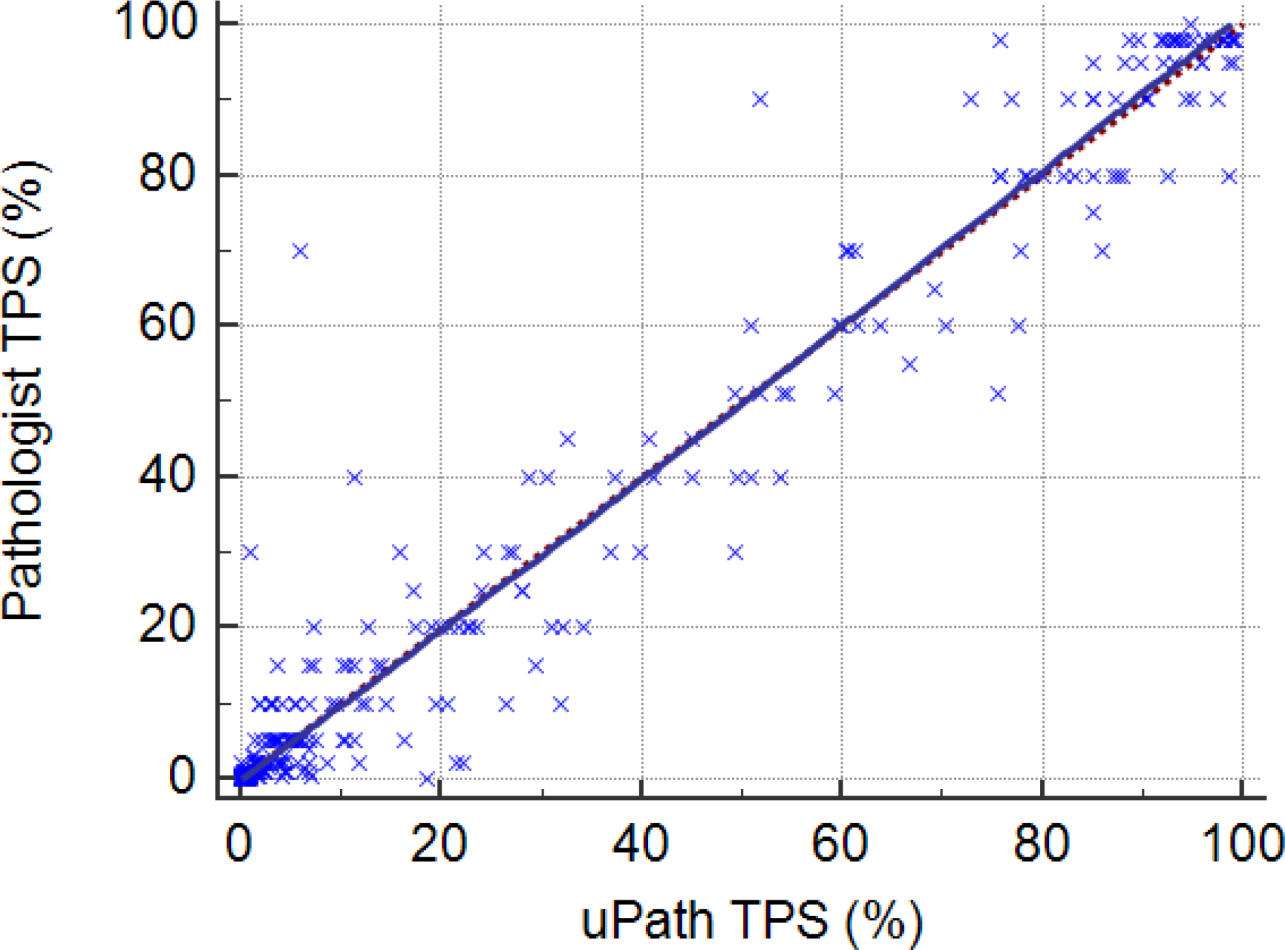
Scatter plot demonstrating high concordance in PD-L1 assessment between uPath and the pathologist (r = 0.945).

In the three-tier classification (<1%, 1–49%, ≥50%), concordance was high, with an overall agreement of 94.5% and a quadratic weighted κ of 0.94 (95% CI: 0.92–0.97). All discrepancies involved only adjacent categories, and no cases were misclassified between <1% and ≥50% (**Table 3**). Continuous TPS values showed a strong positive correlation between methods, with a Spearman’s r of 0.945 (p < 0.001; **Figure 3**).

**Table 3:** Three-tier agreement between uPath and an expert pathologist in PD-L1 TPS categorization. Values represent number of cases (n).

uPath classification	Expert pathologist classification			
	<1%	1–49%	≥50%	Total (n)
<1%	95	11	0	106
1–49%	5	139	2	146
≥50%	0	2	79	81
Total (n)	100	152	81	333

**Figure 4:**
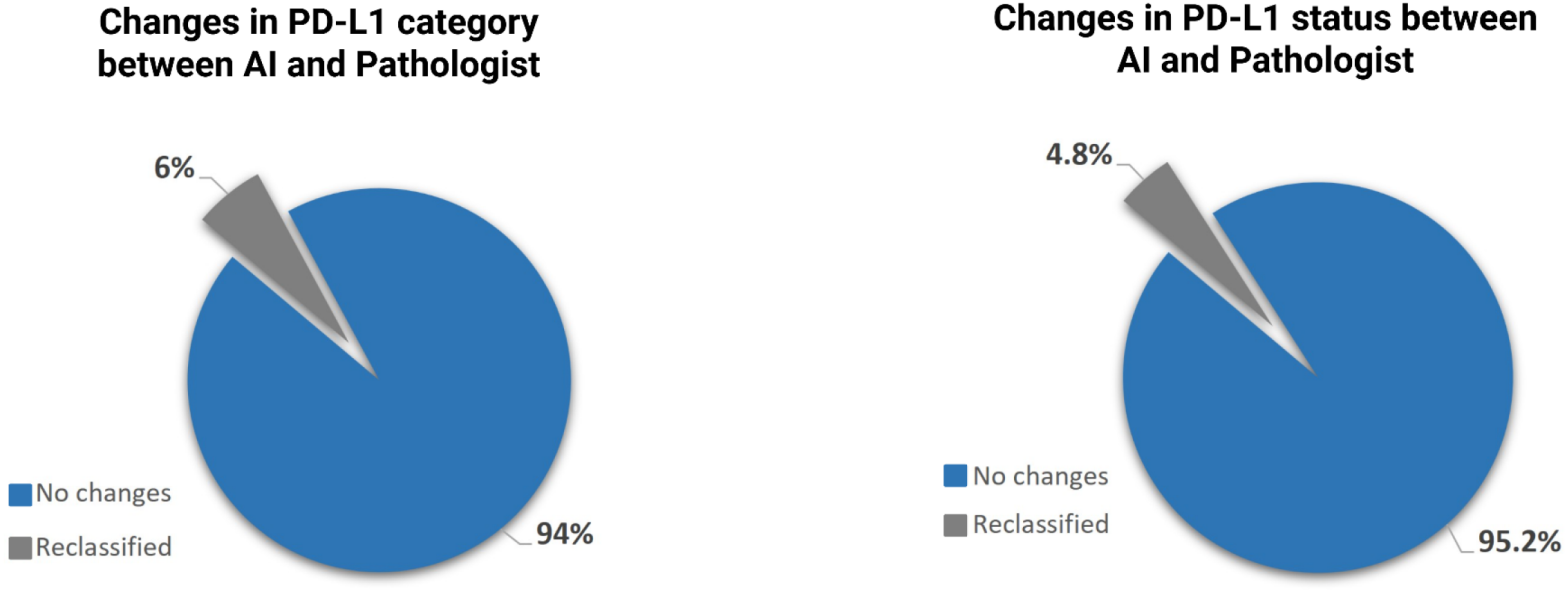
Changes in immunotherapy eligibility based on PD-L1 assessments by uPath versus the pathologist.

The algorithm altered binary PD-L1 status in approximately 4.8% of cases (16/333). Category shifts across TPS strata (<1%, 1–49%, ≥50%) occurred in 6% (20/333), with 13 downgrades and 7 upgrades relative to expert evaluation. When aligned with treatment thresholds, exclusive reliance on AI output would have modified immunotherapy eligibility in about 6% of patients (**Figure 4**).

**Figure 5:**
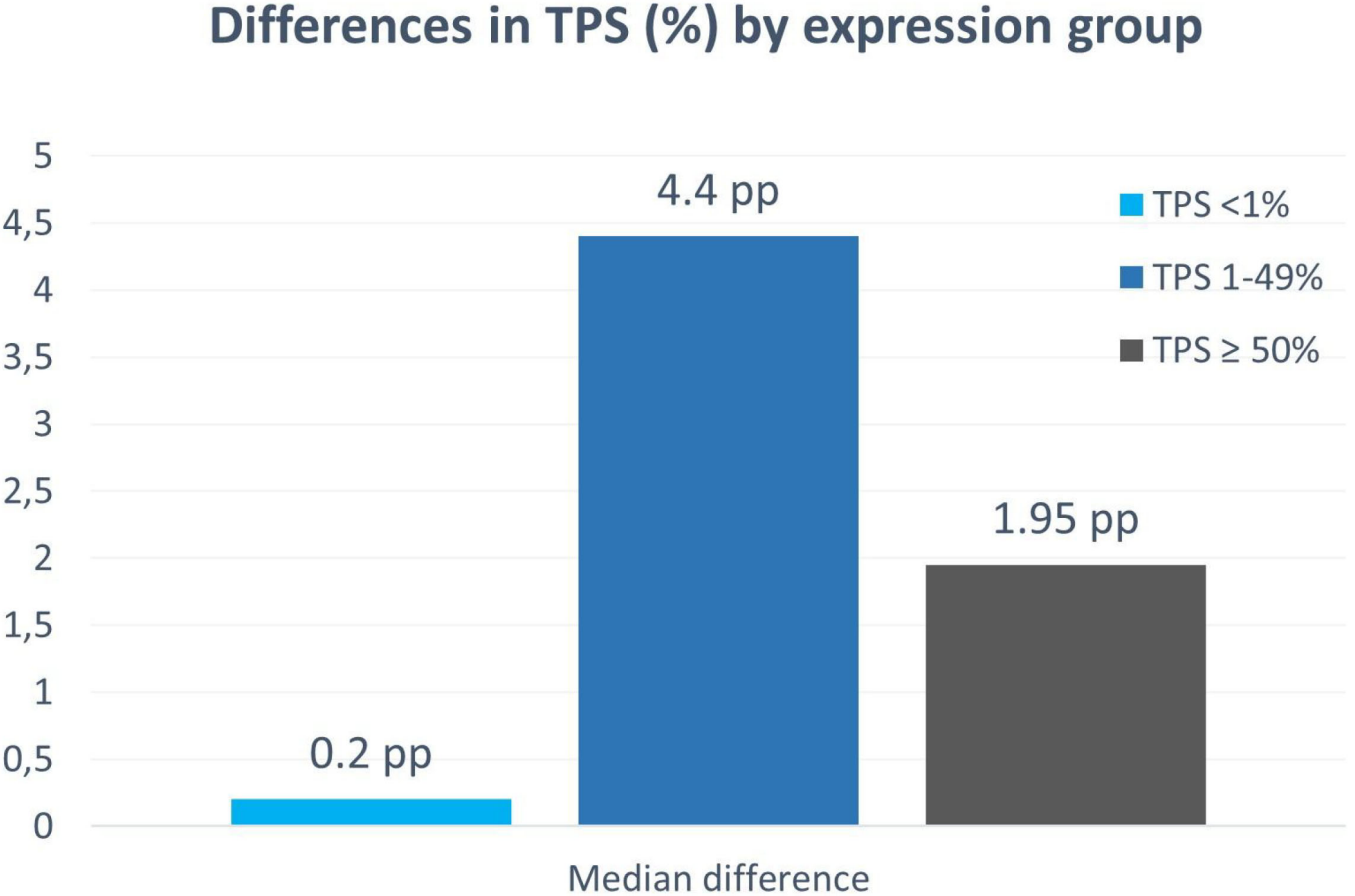
Median TPS differences between uPath and the pathologist, stratified by classification category.

The mean absolute error between algorithm and expert scores was 1.2 percentage points (pp), with a mean difference near zero (≈ 0.0 pp), indicating no systemic bias. Subgroup analyses revealed varying discrepancies by TPS range: mean absolute differences were 0.2 pp for TPS <1%, 4.4 pp for TPS 1–49%, and 1.95 pp for TPS ≥50%. Discrepancies were most pronounced in the intermediate (1–49%) range, where uPath scores tended to exceed the pathologist assessments. Visual inspection of the scatter plot revealed tight clustering along the identity line for high TPS values, with greater but still diagonally centered dispersion in low and intermediate ranges (**Figure 5**).

## 4 Discussion

This study, conducted under real-world clinical conditions, compared conventional and AI-assisted PD-L1 assessments in a pathology department within a Certified European Lung Cancer Centre. The most clinically significant finding was a change in PD-L1 category in 6% (20/333) of cases, potentially affecting treatment decisions. Specifically, the algorithm upgraded 5 cases from PD-L1-negative to positive, downgraded 11 from positive to negative, downgraded 2 from high to positive expression, and upgraded 2 from positive to high expression. These results support the algorithm’s high reproducibility for PD-L1 assessment in NSCLC, with discrepancies lower than the intra- and inter-observer variability reported in the literature (2, 4).

The algorithm’s reproducibility and strong alignment with the gold standard (pathologist assessment) support its use as a secondary tool for borderline cases, particularly in PD-L1-negative (TPS < 1%) scenarios or near the 50% threshold (e.g., TPS 40-49%). This approach could enhance accuracy at low TPS levels (e.g., 0.4-0.9%) and mitigate underestimation near higher cutoffs, consistent with Cortellini et al. (14).

Our findings contrast with some prior reports. Plass et al. examined 51 NSCLC cases and found only fair agreement (Fleiss’ kappa = 0.354) between the pathologists’ consensus and the uPath algorithm (15). Notably, inter-observer agreement in that study was moderate (Fleiss’ kappa = 0.558) for TPS <1% and near-perfect (Fleiss’ kappa = 0.873) for TPS ≥50%, with high intra-observer consistency (Cohen’s kappa ranged from 0.726 to 1.0). In contrast, our algorithm-pathologist agreement (94-95.2%) exceeded their reported inter-observer levels.

Conversely, Cortellini et al. analyzed 44 patients with advanced NSCLC and reported a strong correlation (r = 0.83) between algorithmic and pathologist evaluations (14). However, agreement at the 50% threshold was only 61.4%, suggesting potential diagnostic shifts in 38.6% of cases - substantially higher than the 6% observed in our cohort. Intriguingly, their “uPath high” category correlated with improved overall survival (OS) and progression-free survival (PFS) compared to the “uPath low” group. This illustrates how computational tools - similar to AI-based TROP2 scoring in NSCLC - can yield clinically meaningful insights beyond traditional assessment methods (16).

Most prior studies have focused on the SP263 clone, currently the most widely used antibody in NSCLC diagnostics. Nevertheless, other clinically established clones, such as 22C3, are also used in routine diagnostic practice. Wolf et al. developed an algorithm for automated assessment of both SP263 and 22C3 expression and reported R² values of 0.95 for SP263 and 0.80 for 22C3, indicating superior model performance for SP263 (17). Similarly, Rodriguez et al. reported an overall agreement between manual TPS and HALO PD-L1 Lung AI of 75.4% (95% CI: 0.69–0.81) for the SP263 clone, compared with 72.7% (95% CI: 0.67–0.79) for the 22C3 clone (18). These findings suggest a potentially greater analytical robustness of the SP263 assay in the context of digital image analysis. This discrepancy may be attributed to inherent differences in staining specificity and signal intensity. Despite generally high inter-clone agreement, SP263 typically exhibits stronger staining intensity, albeit with a higher risk of background ‘noise’, whereas 22C3 yields lower intensity with better preservation of cellular morphology. Such subtle immunohistochemical differences may adversely affect algorithm performance and limit the generalizability of AI-based models.

Our department has fully integrated digital pathology into routine histopathological diagnostics, enabling the seamless implementation of advanced computational solutions. Digital workflows allow precise assessment of predictive and prognostic biomarkers, including PD-L1 expression. In our department, AI-assisted image analysis serves as the routine primary modality for PD-L1 scoring. Pathologists review Whole Slide Images (WSIs) integrated with algorithmic assessment outputs and retain the ability to adjust the results when necessary to ensure high-quality assessment. This approach reduces the risk of errors, particularly in borderline cases, and accelerates diagnosis by providing an additional AI-based perspective. Leveraging our position as one of Poland’s leading oncology centers and a Certified European Lung Cancer Centre, we present our observations derived from nearly four years of clinical experience with AI-assisted diagnostic algorithms.

Although AI tools continue to evolve, their strengths in automation must be complemented by expert pathologist oversight, particularly in challenging scenarios such as distinguishing viable tumor cells from macrophages or necrotic areas. The main novelty of this study lies in the direct comparison between an experienced pathologist’s assessment and an AI-assisted scoring, as well as in evaluating the potential influence of these differences on clinical decision-making.

In summary, our findings demonstrate that AI-assisted PD-L1 scoring shows high concordance with expert pathologist assessment and may meaningfully influence clinical decision-making in a subset of cases. Implemented within a real-world digital workflow, the algorithm can serve as a valuable adjunct tool, particularly in borderline scenarios. However, prospective multicenter validation and outcome-based analyses are required to further define its clinical utility and generalizability.

## 5 Limitations

The primary limitation of this study is that PD-L1 TPS was assessed by a single expert pathologist. The study was designed to evaluate algorithm-to-expert concordance rather than interobserver variability, and therefore additional readers were not included. Furthermore, the retrospective single-center design and cohort size, determined by archival WSI availability, may limit generalizability. Clinical diagnostic and treatment decisions for the patients were made prior to this study based on the original pathology reports. Consequently, prospective, multicenter studies with larger cohorts and extended follow-up are needed to validate these results.

## Data Availability

The raw data supporting the conclusions of this article will be made available by the authors, without undue reservation.
